# New clinical and biological insights from the international TARGIT-A randomised trial of targeted intraoperative radiotherapy during lumpectomy for breast cancer

**DOI:** 10.1038/s41416-021-01440-8

**Published:** 2021-05-25

**Authors:** Jayant S. Vaidya, Max Bulsara, Michael Baum, Frederik Wenz, Samuele Massarut, Steffi Pigorsch, Michael Alvarado, Michael Douek, Christobel Saunders, Henrik Flyger, Wolfgang Eiermann, Chris Brew-Graves, Norman R. Williams, Ingrid Potyka, Nicholas Roberts, Marcelle Bernstein, Douglas Brown, Elena Sperk, Siobhan Laws, Marc Sütterlin, Tammy Corica, Steinar Lundgren, Dennis Holmes, Lorenzo Vinante, Fernando Bozza, Montserrat Pazos, Magali Le Blanc-Onfroy, Günther Gruber, Wojciech Polkowski, Konstantin J. Dedes, Marcus Niewald, Jens Blohmer, David McReady, Richard Hoefer, Pond Kelemen, Gloria Petralia, Mary Falzon, David Joseph, Jeffrey S. Tobias

**Affiliations:** 1 Division of Surgery and Interventional Science, University College London, London, UK; 2 Department of Biostatistics, University of Notre Dame, Fremantle, WA Australia; 3 Department of Radiation Oncology, University Medical Center Mannheim, Medical Faculty Mannheim, Heidelberg University, Mannheim, Germany; 4 Department of Surgery, Centro di Riferimento Oncologico di Aviano (CRO) IRCCS, Aviano, Italy; 5 Department of Radiation Oncology, Red Cross Hospital, Technical University of Munich, Munich, Germany; 6 Department of Surgery, University of California, San Francisco, CA USA; 7 Nuffield Department of Surgical Sciences, University of Oxford, Oxford, UK; 8 School of Surgery, University of Western Australia, Perth, WA Australia; 9 Department of Breast Surgery, University of Copenhagen, Copenhagen, Denmark; 10 Department of Gynecology and Obstetrics, Red Cross Hospital, Technical University of Munich, Munich, Germany; 11 Patient advocate and writer, London, UK; 12 Department of Surgery, Ninewells Hospital, Dundee, UK; 13 Department of Surgery, Royal Hampshire County Hospital, Winchester, UK; 14 Department of Gynecology and Obstetrics, University Medical Center Mannheim, Medical Faculty Mannheim, Heidelberg University, Berlin, Germany; 15 Department of Radiation Oncology, Sir Charles Gairdner Hospital, Perth, WA Australia; 16 Department of Oncology, St Olav’s University Hospital, & Department of Clinical and Molecular Medicine, Norwegian University of Science and Technology (NTNU), Trondheim, Norway; 17 John Wayne Cancer Institute & Helen Rey Breast Cancer Foundation, University of Southern California, Los Angeles, CA USA; 18 Department of Radiation Oncology, Centro di Riferimento Oncologico di Aviano (CRO) IRCCS, Aviano, Italy; 19 Department of Surgery, Instituto Oncologico Veneto (IVO) IRCCS, Padoa, Italy; 20 Department of Radiation Oncology, University Hospital, Ludwig Maximilians Universitat, Munich, Germany; 21 Oncologie radiothérapeute, Institut de Cancérologie de l’Ouest, Nantes, France; 22 Brust Zentrum Seefeld, Zurich, Switzerland; 23 Department of Surgical Oncology, Medical University of Lublin, Lublin, Poland; 24 Breast Center, Universitätsspital Zürich, Zurich, Switzerland; 25 Saarland University Medical Center, Homburg, Germany; 26 Sankt Gertrauden-Krankenhaus, and The Charité—Universitätsmedizin Berlin, Berlin, Germany; 27 Princess Margaret Cancer Centre, Toronto, Canada; 28 Sentara Surgery Specialists, Hampton, VA USA; 29 Ashikari Breast Center, New York Medical College, New York, NY USA; 30 Department of Surgery, University College London Hospitals, London, UK; 31 Department of Pathology University College London Hospitals, London, UK; 32 Department of Clinical Oncology, University College London Hospitals, London, UK

**Keywords:** Breast cancer, Radiotherapy, Surgical oncology

## Abstract

**Background:**

The TARGIT-A trial reported risk-adapted targeted intraoperative radiotherapy (TARGIT-IORT) during lumpectomy for breast cancer to be as effective as whole-breast external beam radiotherapy (EBRT). Here, we present further detailed analyses.

**Methods:**

In total, 2298 women (≥45 years, invasive ductal carcinoma ≤3.5 cm, cN0–N1) were randomised. We investigated the impact of tumour size, grade, ER, PgR, HER2 and lymph node status on local recurrence-free survival, and of local recurrence on distant relapse and mortality. We analysed the predictive factors for recommending supplemental EBRT after TARGIT-IORT as part of the risk-adapted approach, using regression modelling. Non-breast cancer mortality was compared between TARGIT-IORT plus EBRT vs. EBRT.

**Results:**

Local recurrence-free survival was no different between TARGIT-IORT and EBRT, in every tumour subgroup. Unlike in the EBRT arm, local recurrence in the TARGIT-IORT arm was not a predictor of a higher risk of distant relapse or death. Our new predictive tool for recommending supplemental EBRT after TARGIT-IORT is at https://targit.org.uk/addrt. Non-breast cancer mortality was significantly lower in the TARGIT-IORT arm, even when patients received supplemental EBRT, HR 0.38 (95% CI 0.17–0.88) *P* = 0.0091.

**Conclusion:**

TARGIT-IORT is as effective as EBRT in all subgroups. Local recurrence after TARGIT-IORT, unlike after EBRT, has a good prognosis. TARGIT-IORT might have a beneficial abscopal effect.

**Trial registration:**

ISRCTN34086741 (21/7/2004), NCT00983684 (24/9/2009).

## Introduction

Most patients with breast cancer are suitable for treatment with breast-conserving surgery and adjuvant radiotherapy, rather than total mastectomy. Based on the hypothesis that adjuvant radiotherapy for women with early breast cancer could be limited to the tumour bed and given immediately during breast-conserving surgery (lumpectomy), we developed the concept of TARGeted Intraoperative radioTherapy (TARGIT-IORT).^1–6^

TARGIT-IORT aims to achieve an accurately-positioned and rapid form of tumour-bed irradiation, focussed on the target tissues alone, sparing normal tissues and organs such as heart, lung, skin and chest wall structures from unnecessary and potentially damaging radiation treatment. We designed the TARGIT-A randomised trial to test this concept by comparing risk-adapted TARGIT-IORT with conventional whole-breast external beam radiotherapy over several weeks (EBRT).^3,7,8^ The study received ethics approval from the Joint University College London and University College London Hospital committees of ethics of human research (99/0307). The accrual was from March 2000 to June 2012. The long-term results of the trial are described separately and show that TARGIT-IORT is as effective as whole-breast external beam radiotherapy (EBRT) for all breast cancer outcomes, with a significant reduction in mortality from causes other than breast cancer.^9^

The trial eligibility was not confined to low-risk patients: they needed to be 45 years or older, with invasive ductal carcinoma that was suitable for breast conservation and preferably less than 3.5 cm in size and unifocal on clinical examination and conventional imaging. Having a grade 3 cancer, involved nodes or higher risk receptor status, did not exclude the patient from participating. Therefore, a large number of patients in each category of higher risk were included, allowing meaningful subgroup analysis. In addition, the follow-up of the TARGIT-A trial was long, with a large number of patients having follow-up for at least 5 years (*n* = 2048) and 10 years (*n* = 741). So, the number of events for local recurrences and deaths after long-term follow-up were expected to be large enough to assess the prognostic significance of local recurrence.

As specified in the protocol, treatment was given using a risk-adapted approach, which meant that patients allocated to receive TARGIT-IORT were recommended to also receive supplemental EBRT, if they were postoperatively found to have specific unsuspected tumour characteristics, in which case the TARGIT-IORT served as a tumour-bed boost. The protocol specified three such factors—an unexpected diagnosis of invasive lobular carcinoma, presence of extensive intraductal component (>25%) and positive margins. Pragmatically, each centre was allowed to pre-specify such criteria and they recorded them in the ‘treatment policy document’ before they started recruitment. Therefore, for an individual case, the use of supplemental EBRT depended on a combination of several factors discussed in the post-operative multidisciplinary team meeting (tumour board). Having known the use of supplemental EBRT within the trial (about 20% of cases) and with the knowledge of the tumour factors, a regression model could be created.

This risk-adapted approach also offers an opportunity for another type of analysis investigating the mechanism of the difference we found in non-breast cancer mortality during the main analysis.^9^ One needs to recognise that the use of supplemental EBRT after TARGIT-IORT was prompted by specific features of the primary breast cancer. Therefore, there should be no reason for the risk of non-breast cancer mortality to be different between patients who received TARGIT + EBRT vs. those who received EBRT. Since both groups received EBRT, and if the difference was because of EBRT toxicity alone, there should be no difference found in non-breast cancer mortality in this comparison.

This paper addresses four important aspects of the trial of TARGIT-IORT vs. EBRT, in which 2298 patients were randomised after their needle biopsy and before any surgical excision of cancer to receive either risk-adapted TARGIT-IORT delivered during the initial excision of cancer, or EBRT. These are: (a) outcome as per well-recognised tumour subgroups, (b) prognostic importance of local recurrence, (c) a predictive model for the use of supplemental EBRT after TARGIT-IORT and (d) an exploration seeking explanation for the differences in non-breast cancer mortality found between the two randomised arms.

## Methods

Data from the TARGIT-A trial (*n* = 2298) comparing risk-adapted TARGIT-IORT given during lumpectomy vs. EBRT were used for these analyses.^9^

The TARGIT-A trial protocol (https://njl-admin.nihr.ac.uk/document/download/2006598), including the details of eligibility, methodology and statistical methods, sample size calculations, the process of random allocation, has been previously described.^7,8,9^ Eligible patients diagnosed with invasive malignancy by needle biopsy were randomly assigned before their surgery, in a 1:1 ratio, to receive either a risk-adapted approach using single-dose TARGIT-IORT or EBRT as per standard schedules over several weeks, with randomisation blocks stratified by centre. Therefore, the trial was a comparison of two policies—whole-breast radiotherapy without selection vs. individualised risk-adapted radiotherapy—in which a proportion of patients who received TARGIT-IORT were also given supplemental EBRT if they were found to have any pre-specified tumour factors.

The sites participating in the trial were all centres of excellence (almost all were University teaching hospitals) with their own routine quality assurance in place. Every patient was treated as per the treatment guidelines and quality assurance laid down by each of the participating radiotherapy centres. While the collection of specific data relating to quality assurance was not mandatory, the schedule of treatment, total dose, dose per fraction and number of fractions for the EBRT (and the boost when given) were always collected. In the UK, the most widely used dose-fractionation regimen recommended during the time of the study was 40.05 Gy/15 fractions over 3 weeks, i.e., daily dose 2.67 Gy per fraction. In the USA, the commonest recommendation was 50 Gy/25 fractions over 5 weeks. For boost doses, institutional standards were once again routinely employed—mostly 10 Gy/5 fractions.

The statistical analysis plan (SAP, submitted with the manuscript) was signed off by the chair of the independent steering committee and an independent senior statistician, before the data were unblinded and  sent to the trial statistician for analysis. It specified the primary outcome as local recurrence-free survival. This outcome measured the chance of a patient being alive without local recurrence (any type of local recurrence in the ipsilateral breast) and therefore included local recurrence or death as events, i.e., patients who had died were not censored, which is consistent with the DATECAN^10^ and STEEP^11^ guidelines for clinical events to be included in the definitions of time-to-event endpoints in randomised clinical trials assessing treatments for breast cancer^12^. All analyses were by intention-to-treat as per the randomisation arm.

Firstly, we performed a subgroup analysis for the primary outcome of local recurrence-free survival for the tumour factors such as size, grade, lymph node involvement, ER status, PgR status and HER2 status.

Secondly, the concern that a difference in local recurrence might increase long-term mortality prompted us to investigate the assumption that local recurrence is a harbinger of distant disease and ultimately of death. We, therefore, performed Cox regression analysis using local recurrence as a time-dependent covariate, and estimated its interaction for the hazards of distant disease, breast cancer mortality in the two randomised arms. We also assessed this for overall mortality in order to take away any bias from the misclassification of the cause of death.

Thirdly, we prepared a regression model using established high-risk factors to predict the use of supplemental EBRT in patients randomised to TARGIT-IORT. Significant factors from the model were used to create an interactive tool that would simulate how patients were treated in the TARGIT-A trial and whether they received supplemental EBRT. Such a tool should help clinicians decide which patients would have received such supplemental EBRT and enable them to translate the risk-adapted approach used within the randomised trial into day-to-day clinical practice.

Finally, we explored the reason for the statistically significant difference in non-breast cancer mortality already seen between the two randomised arms. We compared non-breast cancer mortality between those who had received TARGIT-IORT followed by supplemental EBRT vs. EBRT. Any difference between these two groups would be indicative of a *beneficial* effect of TARGIT-IORT because both groups had received EBRT.

The first patient was randomised in March 2000, and the last in June 2012. The reference date for completeness of follow-up was May 2, 2018. The reference date for analysis was July 3, 2019, so that all events in the entire population up until July 2, 2019 were included for analysis of hazard ratios. Point estimates are given for 5 years, at which point the follow-up is complete, and hazard ratios are estimated for the full length of the follow-up period, i.e., the length of time from randomisation to the date of the latest follow-up, for each individual patient. STATA version 16.0 was used for data compilation, validation and analysis. The chief investigator/corresponding author and the trial statistician had access to all data sent by the trial centre for the analysis; all authors were responsible for the decision to submit the manuscript. Since the last analysis, the trial oversight has been provided by an independent steering committee, appointed by the Health Technology Assessment Programme of the National Institute of Health Research, Department of Health, UK.

## Results

In total, 1140 patients were randomised to TARGIT-IORT and 1158 to whole-breast radiotherapy. Patients were recruited from ten countries (24.7% from UK, 65.1% Europe, 9.4% USA/Canada and 0.8% others). Supplementary Table 1 shows the characteristics of trial patients.

As previously published,^9^ there was no statistically significant difference in local recurrence-free survival (events 167 vs. 147, hazard ratio 1.13, 95% confidence interval 0.91–1.41, *P* = 0.28), distant disease-free survival (133 vs. 148 events, HR 0.88, 0.69–1.12, *P* = 0.30), mastectomy-free survival (170 vs. 175 events, 0.96, 0.78–1.19, *P* = 0.74) or breast cancer mortality (65 vs. 57 events, HR 1.12, 0.78–1.60, *P* = 0.54). There was a significant reduction in non-breast cancer mortality with TARGIT-IORT (45 vs. 74 events, HR 0.59, 0.40–0.86, *P* = 0.005).

In addition, no difference was found in local recurrence-free survival when the following comparisons were made: EBRT patients vs. TARGIT-IORT patients who received additional EBRT (HR 1.19, 0.83–1.71, *P* = 0.3422) and EBRT patients vs. TARGIT-IORT patients who did not receive additional EBRT (HR 1.12, 0.88–1.41, *P* = 0.3661) (Supplementary Fig. 1).

The new analysis presented in this paper examines four specific aspects of the data accrued from this large, randomised trial.

**Fig. 1:**
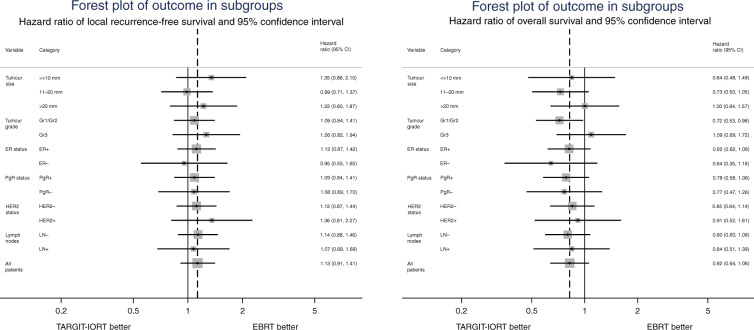
Forest plot showing local recurrence-free survival and overall survival as per tumour subgroups. Each box represents the amount of the data and horizontal lines show the 95% confidence interval. The dashed vertical line is through the hazard ratio for all patients.

Firstly, the difference in the primary outcome of survival without local recurrence between TARGIT-IORT and EBRT was not significant for any of the tumour subgroups viz pathological tumour size, grade, ER status, PgR status, HER2 status and lymph node status (Table 1 and Fig. 1). Prompted by comments from reviewers, we created subgroups using combinations of factors and performed the following analyses. The most substantial of these include 1468 (64%) ‘lower-risk’ patients in whom the tumours were not >2 cm, or grade 3 or ER-negative, irrespective of age or lymph node status (59% were <65 years old and 17% were node-positive). The remaining 830 patients (‘not-lower-risk’) would have at least one of these risk factors.

**Table 1:** Subgroup analysis: number of events for local recurrence and deaths and point estimates for local recurrence-free survival are given for 5 years when the follow-up is complete, as per protocol.

			TARGIT-IORT			EBRT			TARGIT-IORT	EBRT	Long-term local control TARGIT-IORT vs EBRT	
Subgroup	Category	No. of cases	No. of cases	LR	Deaths	No. of cases	LR	Deaths	Alive without local recurrence	Alive without local recurrence	Hazard ratio	95% confidence interval of hazard ratio
Tumour size	<=10 mm	739	369	10	8	370	2	10	94.9%	96.7%	1.35	0.86–2.10
	11–20 mm	1128	571	11	16	557	5	25	95.5%	94.6%	0.99	0.71–1.37
	>20 mm	366	176	2	18	190	3	20	88.5%	88.2%	1.22	0.80–1.80
Tumour grade	Grade 1 or 2	1797	914	17	25	914	7	39	94.9%	96.7%	1.08	0.83–1.40
	Grade 3	443	226	7	17	217	4	17	90.1%	91.1%	1.26	0.82–1.94
ER status	ER+	2035	1005	15	35	1030	9	46	94.9%	94.7%	1.12	0.87–1.42
	ER−	207	114	8	6	105	2	10	89.2%	87.7%	0.95	0.55–1.65
PgR status	PgR+	1816	895	13	29	921	9	40	95.1%	94.7%	1.09	0.84–1.41
	PgR−	413	220	10	12	193	2	16	90.7%	90.9%	1.08	0.69–1.70
HER2 status	HER2−	1845	920	19	35	925	8	39	94.2%	95.0%	1.12	0.87–1.44
	HER2+	320	156	3	6	164	3	16	94.0%	88.7%	1.36	0.81–2.27
Lymph node status	LN−	1765	872	20	27	893	9	42	94.4%	94.4%	1.14	0.88–1.46
	LN+	488	254	4	15	234	2	14	93.0%	93.2%	1.07	0.68–1.70
**Overall**	**All patients**	**2298**	**1140**	**24**	**42**	**1158**	**11**	**56**	**94.2%**	**94.2%**	**1.13**	**0.91–1.41**

*LR* local recurrence.

The hazard ratio for local recurrence-free survival is given for the whole follow-up period and shows that in every subgroup, there was no significant difference in local control (i.e., the probability remaining local recurrence-free) between TARGIT-IORT and EBRT, and 95% CI of the hazard ratio for local recurrence-free survival crossed 1.0, as represented in Fig. 1.

Patients in whom the specific pathological detail was not known were, —for local recurrence: one in each arm for tumour size, in TARGIT-IORT arm 1 ER/PgR status, 2 HER status and, —for death: one in EBRT arm for tumour size, one in TARGIT-IORT arm for ER/PgR/HER2 status.

**Fig. 2:**
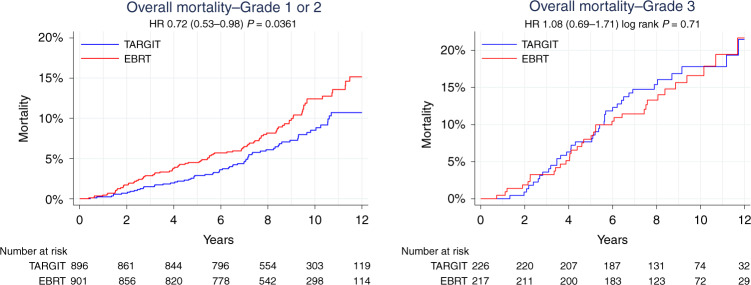
Subgroup analysis: overall survival in those with grade 1 or 2, *n* = 1797, and those with grade 3 cancers, *n* = 443. In total, 80% of the patients had grade 1 or 2 cancers. Of those with grade 1 or 2 cancers vs. grade 3 cancers, 20 vs. 30% were node-positive, and 4 vs. 29% were ER-negative, respectively. There was no difference in the rate of additional EBRT given after TARGIT-IORT between these groups.

Analysis within each of these two subgroups found no difference in local control between the randomised arms TARGIT-IORT vs. EBRT by intention-to-treat, (‘lower-risk’ *n* = 1468, HR 1.05 (95% CI 0.77–1.44, *P* = 0.7450 and ‘not-lower-risk’ *n* = 830, HR 1.24 95% CI 0.91– 1.70, *P* = 0.1715), or after excluding those who received supplemental EBRT after TARGIT-IORT (*n* = 1331, ‘lower-risk’ HR 1.02 (0.73–1.43), *P* = 0.8859 and ‘not-lower-risk’ *n* = 726, HR 1.28 (0.92–1.79), *P* = 0.1404). Similarly, no difference was found for the higher-risk subgroup of with triple-negative breast cancers by intention-to-treat (*n* = 143, HR 0.87 (0.45–1.67), *P* = 0.6840) or after excluding those who received supplemental EBRT (*n* = 131, HR 0.84 (0.43–1.66), *P* = 0.6300)), or those with HER2-negative tumours which were either ER- or PR-negative by intention-to-treat (*n* = 317, HR 1.01 (0.60–1.69), *P* = 0.9730), or after excluding those who received supplemental EBRT (*n* = 281, HR 1.03 (0.60–1.78), *P* = 0.9039). However, for those 1468 ‘lower-risk’ patients (not > 2 cm, or grade 3 or ER-negative), overall survival with TARGIT-IORT was 4.2% better at 12 years (TARGIT-IORT 91.7% vs. EBRT 87.3%, HR 0.65 (95% CI 0.44–0.96), *P* = 0.0308). Figure 1 also shows the overall survival outcomes in each main subgroup. The overall survival was significantly better by 4.4% (89.3 vs. 84.9%) at 12 years with TARGIT-IORT compared with EBRT in those with grade 1 or 2 cancers, (Fig. 2, *n* = 1797, HR 0.72, 95% CI 0.53–0.98, *P* = 0.0361). We recognise of course that these are subgroup analyses, with all the usual caveats.

**Fig. 3:**
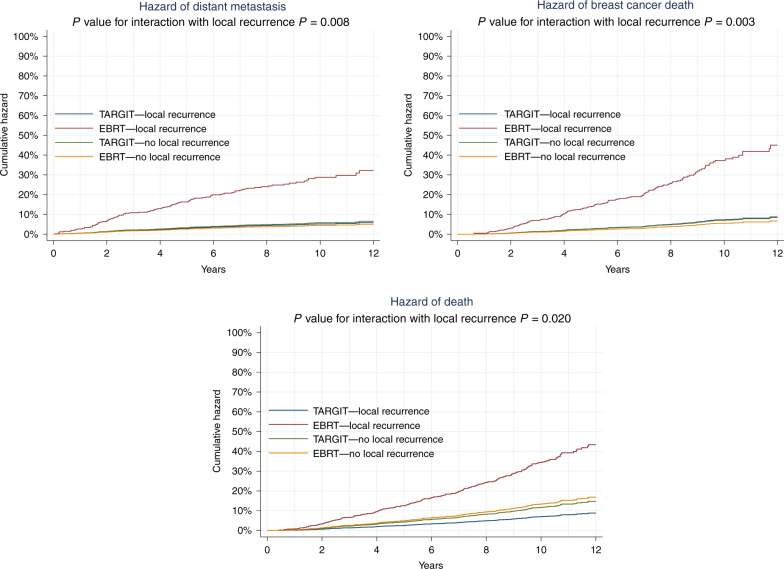
TARGIT-IORT vs EBRT: Contrasting long-term outcome after local recurrence. The hazard of distant metastasis (top left), breast cancer death (top right) and any death (bottom) —interaction with local recurrence as a time-dependent covariate. The hazards of patients who have local recurrence after EBRT as shown by the rising red line in each graph are significantly higher than those who have local recurrence after TARGIT-IORT, which in turn are the same as those without any local recurrence. Please note that these figures denote cumulative hazards of each interaction groups, whereas the curves in Fig. 4 are Kaplan–Meier estimates of cumulative incidences.

Secondly, the analysis of an interaction between local recurrence and mortality found that the prognostic significance of local recurrence in the EBRT arm was different to that of local recurrence in the TARGIT-IORT arm. Local recurrence in the EBRT arm but not in the TARGIT-IORT arm predicted a higher risk of distant disease (*P* value for interaction *P* = 0.008, Fig. 3a), breast cancer mortality (*P* value for interaction *P* = 0.003, Fig. 3b), and overall mortality (*P* value for interaction *P* = 0.020, Fig. 3c). This interaction might be better appreciated when seen in terms of the raw numbers of long-term deaths amongst those who had local recurrence within 5 years: 3/24 (13%) died in the TARGIT-IORT vs. 7/11 (63%) died in the EBRT arm. The mean survival duration of patients who had early local recurrence in the TARGIT arm was 8.7 years (SD 3.1) vs. EBRT 6.1 years (SD 3.3).

Thirdly, the proportion of patients who ultimately received supplemental EBRT in addition to TARGIT-IORT for each prognostic subgroup is given in Table 2, which also gives the local recurrence and mortality events, cumulative incidence of local recurrence, and local control rates as per treatment received. The regression model (sensitivity 71%, specificity 67%, correct classification in 68% of cases) for predicting the use of supplemental EBRT in an individual patient is available on the web and can be best understood with direct interaction. We urge the readers to click on the link https://targit.org.uk/addrt and input some numbers for a hypothetical patient—this way best illustrates the concept—how a combination of factors influence the decision. Two example cases are illustrated in Supplementary Fig. 2. In order to achieve results similar to those achieved within the trial, clinicians would want to emulate the way the risk-adapted approach was used within the trial. This interactive tool gives the probability of any individual patient’s receipt of supplemental EBRT if they had participated in the TARGIT-A trial. Using this information could facilitate an informed decision about recommending supplemental EBRT for an individual patient.

**Table 2:** Total number of patients, total numbers in each arm and proportion of patients receiving supplemental EBRT among those randomised to receive TARGIT-IORT.

		Allocated TARGIT-IORT				Allocated EBRT
	Total no.	Characteristics of 1140 patients in the TARGIT arm	Characteristics of 241 patients allocated TARGIT who received supplemental EBRT	Characteristics of 899 patients allocated TARGIT who did not receive supplemental EBRT	Proportion (%) in TARGIT arm receiving supplemental EBRT	Characteristics of 1158 patients in the EBRT arm
**Overall**	**2298**	**1140**	**241**	**899**		**1158**
*Age (years)*						
≤50	216	117	24	93	20.5%	99
51–60	737	362	81	281	22.4%	375
61–70	1005	481	100	381	20.8%	524
>70	340	180	36	144	20.0%	160
*Tumour size*						
≤10 mm	739	369	58	311	15.7%	370
11–20 mm	1128	571	121	450	21.2%	557
>20 mm	366	176	59	117	33.5%	190
*Grade*						
Grade 1	561	275	42	233	15.3%	286
Grade 2	1236	621	148	473	23.8%	615
Grade 3	443	226	50	176	22.1%	217
*Margins*						
Negative	2000	1007	191	816	19.0%	993
Positive	252	119	49	70	41.2%	133
*Invasive lobular carcinoma at final histology*						
Negative	2112	1053	208	845	19.8%	1059
Positive	120	58	30	28	51.7%	62
*Lymphovascular invasion*						
Absent	1877	931	172	759	18.5%	946
Present	357	185	63	122	34.1%	172
*Nodal status*						
Negative	1765	872	147	725	16.9%	893
1–3 nodes	418	213	76	137	35.7%	205
4 or more	70	41	17	24	41.5%	29
*ER status*						
Positive	2035	1005	218	787	21.7%	1030
Negative	207	114	20	94	17.5%	93
*PgR status*						
Positive	1816	895	191	704	21.3%	921
Negative	413	220	47	173	21.4%	193
*HER2 status*						
Positive	320	156	41	115	26.3%	164
Negative	1845	920	188	732	20.4%	925
*Method of presentation*						
Screen-detected	1494	739	148	591	20.0%	755
Symptomatic	719	364	86	278	23.6%	355
Total number		**1140**	**241**	**899**	**–**	**1158**
Local recurrences (invasive/DCIS/unknown) cumulative incidence		15/6/3 1.3%/0.5%/0.3%	2/1/0 0.8%/0.4%/0%	13/5/3 1.4%/0.6%/0.3%	–	9/1/1 0.8%/0.1%/0.1%
Cumulative incidence of any type of local recurrence		24 2.11%	3 1.24%	21 2.35%		11 0.95%
Deaths (cumulative incidence)		42 (3.7%)	14 (5.8%)	28 (3.1%)	–	56 (4.8%)
Alive without local recurrence		94.15% (92.6–95.4)	93.46% (89.4–96.0)	94.33% (92.6–95.7)	–	94.19% (92.6–94.4)

*LRFS* local recurrence-free survival.

Of the 1140 randomised to TARGIT-IORT, 241 received supplemental EBRT after TARGIT-IORT during lumpectomy. The local recurrence and mortality and local control values are at complete follow-up of 5 years.

**Fig. 4:**
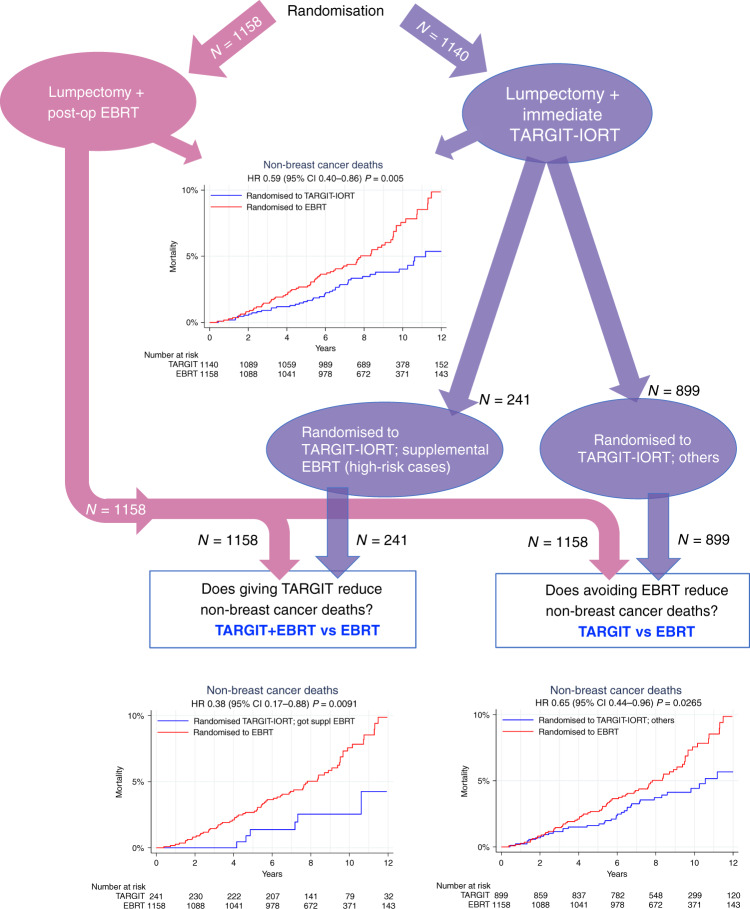
Randomised comparison of non-breast cancer mortality showing signifcantly fewer deaths in patients randomised to TARGIT-IORT(top graph), and non-randomised comparisons to assess the contribution to the difference seen in the randomised comparison: because of the delivery of TARGIT-IORT (bottom left), and the avoidance of EBRT (bottom right). Please note that 40% of patients in the 1158 EBRT arm also received a tumour-bed boost which was not given to those who had received TARGIT-IORT.

Finally, an exploratory analysis sought an explanation for the difference in non-breast cancer mortality that was found in the main analysis between the two randomised arms (HR 0.59 (0.40–0.86), *P* = 0.005). The numbers of non-breast cancer deaths in those who were randomised to TARGIT were 45/1140 (6/241 amongst those who received additional EBRT and 39/899 amongst the others), and 74/1158 amongst those randomised to EBRT. Most of this difference (79% of the difference in the number of deaths) was contributed to by differences in deaths from pulmonary, cardiovascular causes and other cancers. Two of the major risks for these conditions, age and body mass index, were equally distributed in the two randomised arms (Supplementary Table 2, top). Of the 1140 patients randomised to TARGIT-IORT, 241 patients were deemed to have a higher risk of relapse of breast cancer by the treating multidisciplinary team and therefore were selected to receive supplemental EBRT. While this group would have a higher risk of death from breast cancer, they should not have an increased risk of death from non-breast cancer causes—this was corroborated by the well-balanced distribution of two recorded risk factors (age and BMI, Supplementary Table 2, bottom). We found that patients who had TARGIT-IORT plus EBRT (*n* = 241) had a statistically significant reduction in non-breast cancer mortality (HR 0.38 (95% CI 0.17–0.88), *P* = 0.009) when compared with those randomised to EBRT (*n* = 1158), in addition to the significant difference seen in the remaining 899 patients (HR 0.65 (95% CI 0.44–0.96), *P* = 0.0265) (Fig. 4).

## Discussion

The long-term results of the TARGIT-A trial^9^ have shown that there was no statistically significant difference between EBRT and the approach of risk-adapted TARGIT-IORT during lumpectomy, for local recurrence-free survival, invasive local recurrence-free survival, mastectomy-free survival, distant disease-free survival or breast cancer mortality. The mortality from other causes was significantly lower in the TARGIT-IORT arm.

In this paper, we found that the results remain the same for each of the tumour subgroups such that no particular subgroup fares better or worse in terms of the difference in local recurrence-free survival for TARGIT-IORT vs. EBRT. This finding could make it easier for clinicians to select patients. In order to be eligible for risk-adapted TARGIT-IORT, patients simply need to fulfil the eligibility criteria for the TARGIT-A trial (≥45 years of age with invasive ductal carcinoma ≤3.5 cm in size and cN0–N1 and suitable for breast conservation). Once the final histopathology is available postoperatively, the interactive tool based on our regression model could facilitate decision-making about the need for supplemental EBRT: a clinician can input values for characteristics for an individual patient and their tumour in this web-based tool (https://targit.org.uk/addrt), and its output will show the probability that that patient would have received supplemental EBRT after TARGIT-IORT within the TARGIT-A trial. This can help the clinician to make an individualised decision for their patient so that the outcome would be similar to that achieved within the TARGIT-A trial.

An important point that traditionally causes concern is the long-term prognosis of a patient with a local recurrence. A local recurrence has been generally regarded as a harbinger of early death. This idea is supported by the results of the meta-analysis of breast-conserving surgery and whole-breast external beam radiotherapy by the Early Breast Cancer Trialists Collaborative Group,^13^ which determined that for every four additional women who had a local recurrence one died from their disease. Consistent with this long-held belief, the analysis in the TARGIT-A trial presented in this paper also found that a local recurrence after EBRT was indeed a powerful predictor of distant metastases, breast cancer mortality and overall mortality. In contrast, a local recurrence after TARGIT-IORT did not have any impact on distant metastases, breast cancer mortality and overall mortality (Fig. 3). We recognise that the number of events is small, but the statistical significance of this finding is very high (*P* = 0.003). This remarkable finding suggests that local recurrences after TARGIT-IORT are not indicative of the expected poor prognosis that is seen with local recurrences after whole-breast external beam radiotherapy. Possible explanations for this important observation need further research, but some suggestions about its mechanisms are the following:

A simple explanation might be that majority of local recurrences after TARGIT-IORT are new primaries that normally do not have a poor prognosis while EBRT may be suppressing these good-prognosis cancers. The corroboration of this idea is seen in the much higher DCIS: Invasive ratio (12:32 vs. 1:19) in the TARGIT-IORT arm compared with EBRT, raising the possibility of overdiagnosis and ascertainment bias because of potentially more frequent use of mammography in those randomised to TARGIT-IORT. This may have led to a higher chance of detection of DCIS or invasive cancers that may not have progressed. However, this detection of such good-prognosis cancers in the TARGIT-IORT arm did not cause any reduction in mastectomy-free survival. We might also speculate that after EBRT, a local recurrence has only very aggressive cells that are a marker of incurable distant disease or consist of metastatic cells that grow in the tumour supportive wound environment. TARGIT-IORT appears to favourably influence wound fluid composition, and this may be a mechanism by which it might have unique radiobiology that somehow mainly allows the expression of local recurrences that are curable by earlier surgery and change of systemic (usually endocrine) therapy.

By corollary, one might argue that avoiding radiotherapy altogether might have even enhanced such effect—but randomised evidence tells us that it does not—in trials of EBRT vs. no EBRT, for every four local recurrences that occur in the absence of EBRT there is one additional death^13^. So TARGIT-IORT may be stopping the growth of local recurrences that have the potential to spread and cause death, whilst allowing those local recurrences that are a marker of curable distant disease to grow and raise an early flag just like the canary in the coal mine. Further research comparing the molecular characteristics of local recurrences between the two arms of the trial could give more insight into the biological nature of these recurrences.

The other striking outcome in the trial was that there was a significant reduction in non-breast cancer mortality in patients randomised to TARGIT-IORT. Now, within those randomised to TARGIT-IORT, there were some patients (*n* = 241) who also had received supplemental EBRT because they had a higher risk of breast cancer relapse. However, their risk of non-breast cancer death should not be any different from those who were randomised to EBRT. Surprisingly, there was a statistically significant difference in non-breast cancer mortality (HR 2.62 (1.14–6.04), *P* = 0.0093) between them, and those allocated to EBRT. As both these groups received EBRT, the reduction in non-breast cancer mortality cannot be attributed to the absence of EBRT, but rather must be attributed to the presence of TARGIT-IORT. There is however one caveat—40% of those in the EBRT arm also received a tumour-bed boost (reminding us that TARGIT-A was a medium-risk cohort), so this higher dose may have contributed to the effect. In any case, the baseline major risk factors for these deaths (age and BMI) were well balanced between these non-randomised groups (Supplementary Table 1). This long-term outcome is consistent with previous reports and prompts the hypothesis that a single large dose of radiation such as TARGIT-IORT given during the trauma of surgery might possibly have an abscopal effect, i.e., an effect away from the site of irradiation, by influencing the tumour microenvironment or by immunological mechanisms.^14–27,28^ Strange as it may seem, such an abscopal effect appears to give long-term protection against deaths from cardiovascular causes and other cancers. The early separation of lines in the K–M curves that starts soon after randomisation also suggests such a ‘drug-like’ effect, while a separation starting a few years later in the comparison of TARGIT-IORT alone vs. EBRT suggests an effect of avoiding EBRT (Fig. 4). We believe that for the effect of immediate TARGIT-IORT on wound fluid, and its potential abscopal effects, the temporal proximity of TARGIT-IORT to surgery is crucially important. This TARGIT-IORT delivery to the fresh tumour bed immediately after lumpectomy, without any additional trauma, did not happen in the delayed IORT trial.^29^ The IORT in the experimental arm in that separate study^29^ was delivered at a median of 37 days postoperatively, by re-opening the wound. This difference in timing of radiotherapy may well offer an explanation for the difference in non-breast cancer mortality outcomes. Of course, we need to recognise that these data only generate the hypothesis, and do not prove an abscopal effect. The TARGIT-B superiority trial, in which patients are being recruited from 38 centres in 15 countries, is comparing TARGIT-IORT boost during lumpectomy, in addition to post-operative whole-breast radiotherapy, vs. conventional EBRT (i.e., TARGIT-IORT + EBRT vs. EBRT). It will provide randomised data to assess such putative abscopal effects.

In conclusion, these long-term data from the TARGIT-A trial show that for every subgroup of patients with breast cancer who meet our trial selection criteria, risk-adapted single-dose TARGIT-IORT during lumpectomy is an effective and safe alternative to several weeks’ course of post-operative EBRT. The observation that local recurrence after TARGIT-IORT, unlike after EBRT, does not have a poor prognosis is reassuring. The potential beneficial effect of TARGIT-IORT during surgery on non-breast cancer mortality seen in this trial has increased the importance of forthcoming randomised data on non-breast cancer mortality from the TARGIT-B trial.

## Supplementary information

CONSORT checklist

Supplemental information

## Data Availability

Not applicable.

## Data sharing statement

UCL is supportive of data sharing and will endeavour to assist in requests for data sharing. All requests for data sharing will adhere to the UCL Surgical & Interventional Trials Unit (SITU) data-sharing agreement policy. These data will be held at UCL on secure servers and cannot be released to any third parties. All requests for access to the data will be formally requested through the use of a SITU data request form which will state the purpose, analysis and publication plans together with the named collaborators. All requests are dealt with on a case by case basis. All requests will be logged and those successful will have a data transfer agreement which will specify appropriate security and privacy agreements, and acknowledgement of the TARGIT Trialists’ Group, investigators, the sponsor and funders.
