# Supplementary material for: New clinical and biological insights from the international TARGIT-A randomised trial of targeted intraoperative radiotherapy during lumpectomy for breast cancer
Source: Br J Cancer. 2021 May 25;125(3):380–9. doi: 10.1038/s41416-021-01440-8 (PMC8329051; doi:10.1038/s41416-021-01440-8)
Supplement: Supplementary file 2 — Supplemental information [file 41416_2021_1440_MOESM2_ESM.docx]

**Supplementary figure 1**

Kaplan-Meier plot of local recurrence-free survival of those randomised to receive EBRT (red line) along with those randomised to receive TARGIT-IORT separated by those who received additional EBRT (purple line) and those who did not (blue line). No statistically significant difference was found between EBRT and the two latter groups.

**Supplementary figure 2 –** An illustration of the tool to assess which patient we recommend EBRT after TARGIT-IORT based on the data from the TARGIT-A trial**.** *We urge the readers to please click on the link* <https://targit.org.uk/addrt> – *and input some numbers for a hypothetical patient to best illustrate the concept.*

**
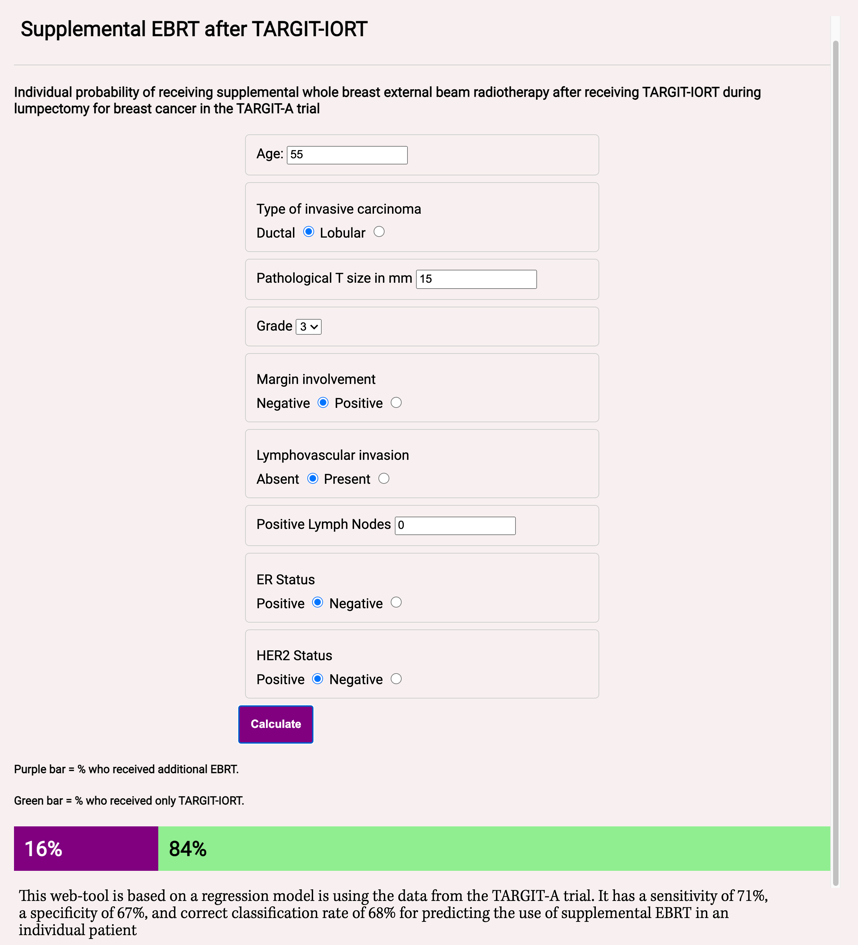
**

**
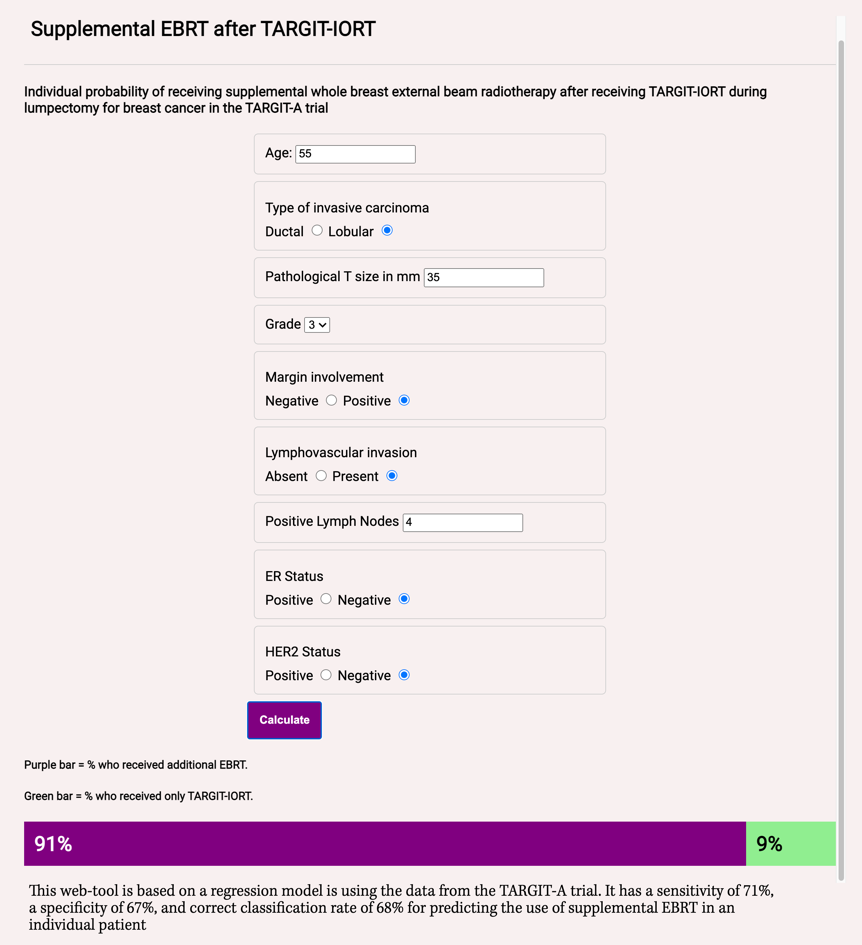
**

**Supplementary table 1 – Patient and tumour characteristics**

| **Characteristics** | **TARGIT-IORT (n=1140)** | **EBRT (n=1158)** |
| --- | --- | --- |
| **Age (years)** | | |
| ≤50 | 117 (10.3) | 99 (8.6) |
| 51-60 | 362 (31.8) | 375 (32.4) |
| 61-70 | 481 (42.2) | 524 (45.3) |
| >70 | 180 (15.8) | 160 (13.8) |
| **Body mass index** | | |
| Normal (<25) | 408 (41.0) | 420 (42.2) |
| Overweight (25-29.9) | 375 (37.7) | 329 (33.1) |
| Obese (≥30) | 212 (21.3) | 246 (23.7) |
| Unknown | 145 (12.7) | 163 (14.1) |
| **Specimen weight (g)** | | |
| Median (interquartile range) | 40 (25-65) | 40 (24-70) |
| **Pathological tumour size (mm; P=0.70)** | | |
| ≤10 | 369 (33.1) | 370 (33.1) |
| 11-20 | 571 (51.2) | 557 (49.9) |
| >20 | 176 (15.8) | 190 (17.0) |
| Unknown | 24 (2.1) | 41 (3.5) |
| **Grade** | | |
| 1 | 275 (24.5) | 286 (25.6) |
| 2 | 621 (55.4) | 615 (55.0) |
| 3 | 226 (20.1) | 217 (19.4) |
| Unknown | 18 (1.6) | 40 (3.5) |
| **Margin** | | |
| Free | 1007 (89.4) | 993 (88.2) |
| Ductal carcinoma in situ only | 54 (4.8) | 60 (5.3) |
| Invasive | 65 (5.8) | 73 (6.5) |
| Unknown | 14 (1.2) | 32 (2.8) |
| **Re-excision** | 76 (6.7) | 97 (8.4) |
| **Lymphovascular invasion** | | |
| Absent | 931 (83.4) | 946 (84.6) |
| Present | 185 (16.6) | 172 (15.4) |
| Unknown | 24 (2.1) | 40 (3.5) |
| **Lymph nodes involved** | | |
| 0 | 872 (77.4) | 893 (79.2) |
| 1-3 | 213 (18.9) | 205 (18.2) |
| >3 | 41 (3.6) | 29 (2.6) |
| Unknown | 14 (1.2) | 31 (2.7) |
| **Estrogen receptor status** | | |
| Positive | 1005 (89.8) | 1030 (91.7) |
| Negative | 114 (10.2) | 93 (8.3) |
| Unknown | 21 (1.8) | 35 (3.0) |
| **Progesterone receptor status** | | |
| Positive | 895 (80.3) | 921 (82.7) |
| Negative | 220 (19.7) | 193 (17.3) |
| Unknown | 25 (2.2) | 44 (3.8) |
| **Human epidermal growth factor receptor 2 status** | | |
| Positive | 156 (14.5) | 164 (15.1) |
| Negative | 920 (85.5) | 925 (84.9) |
| Unknown | 64 (5.6) | 69 (6.0) |
| **Method of presentation** | | |
| Screen detected | 739 (67.0) | 755 (68.0) |
| Symptomatic | 364 (33.0) | 355 (32.0) |
| Unknown | 37 (3.3) | 48 (4.2) |
| **Endocrine therapy** | | |
| Received | 897 (81.5) | 894 (81.1) |
| Did not receive | 204 (18.5) | 209 (18.9) |
| Unknown | 39 (3.4) | 55 (4.8) |
| **Chemotherapy** | | |
| Received | 239 (21.7) | 218 (19.7) |
| Did not receive | 863 (78.3) | 887 (80.3) |
| Unknown | 38 (3.3) | 53 (4.6) |

EBRT=external beam radiotherapy; TARGIT-IORT=targeted intraoperative radiotherapy.

Data are numbers (percentages). For percentage calculation, the denominator for unknown percentages is the total number randomised (1140 and 1158) and the denominator for each category is the total number of known patients. No imbalance was found for any of these characteristics between the two randomised arms.

**Supplementary table 2 Background risk factors for non-breast-cancer deaths**

|  | **TARGIT-IORT (1140)** | | **EBRT (1158)** | |
| --- | --- | --- | --- | --- |
| Age (Median age is 63) |  | | | |
|  |  |  |  |  |
| <63 years | 572 | 50.18% | 578 | 49.91% |
| >=63 years | 568 | 49.82% | 580 | 50.09% |
| Body Mass Index (BMI) |  | |  | |
| (Normal <25) | 408 | 41.01% | 420 | 42.21% |
| Overweight /Obese (>=25) | 587 | 58.99% | 575 | 57.79% |
|  |  | |  | |

|  | **Patients at higher risk of breast cancer relapse and given TARGIT-IORT plus**  **supplemental EBRT (241)** | | **EBRT (1158)** | |
| --- | --- | --- | --- | --- |
| Age (Median age is 63) |  | | | |
|  |  |  |  |  |
| <63 years | 120 | 49.79% | 578 | 49.91% |
| >=63 years | 121 | 50.21% | 580 | 50.09% |
| Body Mass Index (BMI) |  | |  | |
| Normal (<25) | 85 | 40.09% | 420 | 42.21% |
| Overweight /Obese (>=25) | 127 | 59.91% | 575 | 57.79% |
|  |  | |  | |
